# Management of caustic pharyngoesophageal injuries in Korle Bu Teaching Hospital, Accra, Ghana: a 12-year retrospective review of 29 cases

**DOI:** 10.11604/pamj.2022.42.213.30206

**Published:** 2022-07-19

**Authors:** Mark Tettey, Frank Edwin, Martin Tamatey, Kelechi Okonta, Kow Entsua-Mensah, Innocent Adzamli, Ernest Aniteye

**Affiliations:** 1 University of Ghana Medical School, University of Ghana, Accra, Ghana; 2 National Cardiothoracic Centre, Korle Bu Teaching Hospital, Accra, Ghana; 3 School of Medicine, University of Health and Allied Sciences, Volta Region, Ghana; 4 Department of Surgery, Cardiothoracic Unit, University of Port Harcourt, Choba, Nigeria

**Keywords:** Caustic, pharyngoesophageal, injuries, pharyngoesophagoplasty, colopharyngoplasty

## Abstract

**Introduction:**

caustic pharyngoesophageal strictures are life-threatening injuries with important management difficulties, lacking clear therapeutic guidelines. The aim of this study is to evaluate the surgical procedures and outcomes of severe caustic pharyngoesophageal strictures in our institution.

**Methods:**

a total of 29 patients who underwent surgery for severe caustic pharyngoesophageal injury at the National Cardiothoracic Center from June 2006 to December 2018 were retrospectively reviewed. The age distribution, sex, management procedures, complications after surgery, and the outcome were analyzed.

**Results:**

there were 17 males. The mean age was 11.7 years (range 2- 56 years). All patients accidentally swallowed caustic soda, except the oldest patient who ingested an unidentified substance. The treatment procedures included colopharyngoplasty in 15 (51.7%) patients, colon-flap augmentation pharyngoesophagoplasty (CFAP) in 10 (34.5%), and colopharyngoplasty with tracheostomy in 4 (13.8%). There was one case of graft obstruction from a retrosternal adhesive band and one case of postoperative reflux with nocturnal regurgitation. No cervical anastomotic leak occurred. Rehabilitative training for oral feeding was required for less than a month in most patients. Follow-up period ranged from one to twelve years. Four patients died within this period; two were immediate post-operative deaths and two occurred late. One patient was lost to follow-up.

**Conclusion:**

outcome of surgery for caustic pharyngoesophageal stricture is satisfactory. Colon-flap augmentation pharyngoesophagoplasty reduces the need for tracheostomy before surgery, and our patients start eating early without aspiration.

## Introduction

Caustic pharyngoesophageal stricture presents a daunting task to the clinician who is faced with a complexity of surgical procedures to address life-threatening cicatrization of the upper aero-digestive tract [1]. In those with the severest injuries, a feeding gastrostomy or jejunostomy with or without a tracheostomy would have been fashioned in the initial resuscitative efforts. In developing countries, improper handling of caustic agents used in local soap manufacturing predisposes children to accidental ingestion [2]. These children are often exposed to very high concentrations of caustic agents when ingested, with devastating consequences. Long-term complications of strictures involving the pharynx are life-threatening. Pharyngeal involvement in diffuse caustic upper gastrointestinal stricture is an infrequent yet potentially life-threatening complication reported in 0.7-6.0 % of patients with caustic ingestion [3]. There is currently no gold standard treatment procedure for the management of severe pharyngo esophageal injuries [4]. Institutions generally tailor their management to the severity and extent of involvement of the larynx, pharynx, and esophagus. Even though the airway is essential for survival, this is sometimes sacrificed in selected patients by creating an alternative source of breathing using tracheostomy [5]. In resource-poor countries, there exists a challenge in the management of tracheostomy tubes in patients who undergo colopharyngoplasty [1]. Colon-flap augmentation pharyngoesophagoplasty (CFAP) is an improvement on the already described colopharyngoplasty and allows for successful construction of hypopharynx and neohypopharynx with minimal aspiration when the patient swallows [6]. The challenge for the surgeon is to do the best possible and restore near-normal life after severe pharyngoesophageal injuries. This study seeks to evaluate the outcome of surgical reconstruction of the upper aero-digestive tract in patients with severe intractable pharyngoesophageal strictures.

## Methods

**Study design and setting:** referred to the National Cardiothoracic Center, Accra on account of severe caustic pharyngoesophageal injury from June 2006 to June 2017 were retrospectively reviewed. There were 29 patients in the study.

**Study population:** all patients were referred after undergoing initial feeding gastrostomy during the acute phase of injury. Four patients with severe upper airway stricture had prior tracheostomies. Patients were nutritionally rehabilitated for at least 6 months prior to definitive surgery. Principal investigative modalities used prior to surgery included upper gastrointestinal endoscopy and barium swallow when these were possible.

**Data collection and analysis:** their medical records in the institutional database were reviewed for demographics, operative procedure, and outcome. Data were collected and entered into in spreadsheet and subsequently analyzed to obtain frequencies and proportions.

**Ethical considerations:** the approval to publish the data was granted by the Ethical and Protocol review committee of the College of Health Sciences, University of Ghana. Identification number: CHS-Et/M.4 -4.9/2020-2021

## Results

**General characteristics:** there were 29 patients; 17 were males. Their ages ranged from 2 to 56 years, with a mean age of 11.7 years. The age distribution is shown in Table 1. All patients accidentally ingested caustic soda except the oldest patient who swallowed an unidentified substance.

**Table 1:** age distribution

Age (years)	Frequency	Percentage
0-5	13	44.8
6-10	8	27.6
11-15	0	0
16-20	4	13.8
>20	4	13.8
Total	29	100

**Treatment:** the surgical procedures used (Table 2) included colopharyngoplasty with or without tracheostomy and colon-flap augmentation pharyngoesophagoplasty (CFAP) with or without tracheostomy. Except for one case who had tracheostomy intraoperatively before the colopharyngoplasty to secure the airway, four had permanent tracheostomy before referral to the center.

**Table 2:** operative procedures

Procedure	Number	Percentage
Colopharyngoplasty	15	51.7
Colopharyngoplasty with tracheostomy	4	13.8
Colon-flap augmentation pharyngoesophagoplasty	9	31.0
Colon-flap augmentation pharyngoesophagoplasty with tracheostomy	1	3.5
Total	29	100

**Outcomes:** out of the 10 patients who underwent CFAP, eight of these could swallow satisfactorily within two weeks of hospital discharge without aspiration. One of the two patients who could not swallow within two weeks after CFAP (a two-year-old boy) was referred with a permanent tracheostomy and was able two swallows satisfactorily by six weeks after discharge from the hospital. The tenth patient who underwent CFAP (an 8-year-old boy) required nearly two months to swallow normally without aspiration. Table 3 shows the number of weeks after discharge, our patients were able to swallow without aspiration. There was one case of graft obstruction from a retrosternal adhesive band. Transient mild aspiration with coughing in the initial 2-3 days of postoperative attempts at deglutition was experienced by nearly all patients. Rehabilitative training for oral feeding was required for less than a month in most patients. There was a child with significant reflux after surgery, which regressed spontaneously within two months of discharge. Follow-up period ranged from 1 to 11 years. Four patients died within this period; two were immediate postoperative deaths and two occurred late. One patient was lost to follow-up.

**Table 3:** number of weeks patients were able to swallow after discharge from hospital

Number of weeks	Frequency	Percentage
2	13	44.8
4	7	24.1
6	1	3.5
8	7	24.1
12	1	3.5
Total	29	100

## Discussion

We analyzed 29 patients who presented at our hospital with severe caustic pharyngoesophageal injuries and were treated with surgical repair. Most of the patients treated within the period were children. The procedures used included colopharyngoplasty in 15 (51.7%) patients, CFAP in 10 (34.5%) patients and colopharyngoplasty with tracheostomy in 4 (13.8%) patients. The repair procedure adopted appear to reduce the need for tracheostomy before surgery in patients with preserved larynx after caustic pharyngoesophageal injury. As observed by Contini and coworkers, children represent 80% of the corrosive gastrointestinal tract injury population globally [7]. Among patients with severe pharyngoesophageal strictures in our study, more than 75% of them were 10 years old or younger. The study by Botwe and coworkers demonstrated that most of these victims are children of mothers who engage in domestic soap manufacturing or children of close friends of these mothers [2]. Caustic soda, mostly in the form of concentrated solution left in familiar soft drink containers are mistaken for drinking water by unsuspecting children. The high concentration of these solutions accounts for the severity of pharyngoesophageal injuries seen in the victims. In many developing countries where legislative control of access to caustic substances is nonexistent, preventive efforts are necessarily aimed at educating domestic soap makers on the proper handling and disposal of left-over caustics to avoid inadvertent ingestion [2]. The patients treated in this study presented with varying degrees of strictures involving the oropharynx and the hypopharynx. Management difficulties arise from the challenges of restoring swallowing while preserving the airway. The involvement of the hypopharynx and oropharynx underpin the complexity of any potential procedure to restore the integrity of the aerodigestive tract. As indicated by Nilakantan and coworkers, the swallowing mechanism is often compromised by fibrosis of the soft palate and pharynx with loss of coordination of the pharyngeal muscles and narrowing of pharyngeal space behind the larynx [8]. This is one of the reasons why postoperative deglutitive training to prevent aspiration is often required after pharyngoesophagoplasty. Different procedures have been used over the years to help restore near normal deglutition, but none has so far been accepted as the standard. In the setting of strictures involving the pharynx and hypopharynx, we have found the creation of a neohypopharynx behind the larynx very useful. This affords enough space behind the larynx after repair and reduces laryngeal soiling during swallowing [4,6].

Colopharyngoplasty (with or without tracheostomy) and colon flap augmentation pharyngoesophagoplasty (with or without tracheostomy) were the mainstays of surgical management in this study. The details of these procedures are described elsewhere [1,6]. Two patients died from tracheostomy related complications. Patients with severe caustic injury and cicatrization of the larynx will need a permanent tracheostomy. Colopharyngoplasty in these patients poses no danger of aspiration during swallowing. The difficulty arises when the larynx is spared in the setting of severe pharyngoesophageal stricture [9,10]. The repair is often complicated by aspiration during swallowing and sometimes it takes several months to train patients to swallow without aspiration and occasionally this may not be successful. The challenge in resource-constrained settings is caring for the tracheostomy tube. Such constraints are circumvented by the avoidance of tracheostomy whenever possible. The CFAP turned out to be a valuable option in patients with severe pharyngoesophageal stricture who would have otherwise needed a tracheostomy with just a colopharyngoplasty. CFAP augments the retropharyngeal space by enlarging the oropharynx, hypopharynx, and proximal esophagus using a flap created out of the proximal colon graft to eliminate the narrowing resulting from the stricture [6]. This procedure does not require the step-down procedures adopted by some authors to avoid tracheostomy [10,11]. For fear of aspiration, some workers avoided surgery on patients with no demonstrable lumen in the cervical esophagus [4]. By contrast, most patients in our series who benefited from CFAP had near-total obliteration of the lumen of the cervical esophagus. The 2-year-old boy in this series had severe pharyngoesophageal stricture underwent a permanent tracheostomy prior to referral to our Center. Intraoperatively, the laryngeal opening was almost completely closed, leaving just a pinhole opening. The adhesive closure of the larynx was opened with ease, exposing the vocal cords. A CFAP was performed. Accidentally, the tracheotomy got dislodged at night on the fourth postoperative day. This was not noticed immediately because the child was breathing normally. He was assessed and did not need reintroduction of the tracheostomy tube. This child could swallow normally without aspiration by six weeks after discharge from the hospital. His feeding gastrostomy was kept for an additional 4 weeks, beyond which time it became unnecessary.

Colopharyngoplasty was attended by few complications; transient minor aspiration within the first few days of deglutition was notable. Aspiration was more frequent early in the series, but subsequent patients experienced minor degrees less frequently. This has been particularly significant in those with severe strictures who underwent CFAP most of whom could swallow without significant aspiration 2 weeks after discharge from the hospital. The 8-year-old who could not swallow by 8 weeks after CFAP underwent an esophagoscopy to evaluate the cervical anastomosis. Anastomotic stricture was suspected but not found. Surprisingly, the boy could swallow without aspiration after the esophagoscopy and be discharged home the following day. Rehabilitative training for deglutition without aspiration for most patients was achieved in less than a month. One case of graft obstruction from a retrosternal adhesion band occurred 12 months after surgery. The patient presented with vomiting and regurgitation associated with weight loss. A barium swallow revealed a retrosternal obstruction of the colon. The colon graft was exposed through a mini-sternotomy and the adhesive band was released with symptom resolution after the procedure. The causes of conduit obstruction seen in other studies have been colo-gastric anastomotic stricture, redundant colon, obstruction at the hiatus, and obstruction at the level of the sternum [12,13]. There was one case of significant reflux after CFAP. The child complained of vomiting at night when recumbent but not during the day. Imaging studies revealed no structural defects that could account for the symptom. We conjectured that the colo-gastric anastomosis performed relatively close to the antrum was responsible for gastrocolic reflux. With proper positioning at bedtime (left side down with torso elevated), nocturnal vomiting subsided and ultimately disappeared. Cervical anastomotic leak, a usually common complication, did not occur in this series. Four patients died: two of them from tracheostomy related complications. The third patient, who also had a permanent tracheostomy, died from complications of varicella zoster. The last patient who was a child died at home one year after surgery. The cause of death was not known. The number of cases operated using the CFAP was only ten. We believe as more cases are done using this procedure, it will help support the suitability of this method to repair very severe pharyngoesophageal injuries.

## Conclusion

The outcome of surgical reconstruction of the upper aerodigestive tract following severe caustic strictures is encouraging, with the restoration of deglutition without significant aspiration in the majority. Permanent tracheostomy when required portends a worse prognosis attributable to complications of managing the airway on an out-patient basis. Pharyngoesophagoplasty can be safely performed in most patients, with acceptable outcomes. Colon flap augmentation pharyngoesophagoplasty improves the functional outcome of patients with severe intractable pharyngoesopgahgeal caustic injuries and should be considered an alternative, especially in those in whom colopharyngoplasty with a permanent tracheostomy is being contemplated.

### What is known about this topic

- Repair of high caustic pharyngoesophageal stricture is challenging and patients who undergo this procedure take long periods to learn swallowing without aspiration;
- In patients with high caustic pharyngoesophageal stricture with functional larynx, tracheostomy is necessary before repair to prevent aspiration in the immediate postoperative period.

### What this study adds

- The use of the new procedure termed Colon-flap augmentation pharyngoesophagoplasty (CFAP), aspiration after surgery is minimal and most patient start eating normally within two weeks after discharge from hospital;
- In our series of patients, tracheostomy was not needed before surgery when using colon-flap augmentation pharyngoesophagoplasty.
